# Increased expression of GPX4 promotes the tumorigenesis of thyroid cancer by inhibiting ferroptosis and predicts poor clinical outcomes

**DOI:** 10.18632/aging.204473

**Published:** 2023-01-09

**Authors:** Huanjie Chen, Fang Peng, Jingchao Xu, Guangzhi Wang, Yongfu Zhao

**Affiliations:** 1 Department of General Surgery, The Second Hospital of Dalian Medical University, Dalian, Liaoning Province, People’s Republic of China; 2 Department of Pathology, The Second Hospital of Dalian Medical University, Dalian, Liaoning Province, People’s Republic of China

**Keywords:** GPX4, thyroid cancer, prognostic biomarker, clinicopathological features, ferroptosis

## Abstract

Background: Ferroptosis plays a critical role in suppressing cancer progression, and its essential regulator is glutathione peroxidase 4 (GPX4). High GPX4 expression can inhibit accumulation of iron, thus suppressing ferroptosis. However, its function in thyroid cancer has not been fully illuminated. Here, we explore the effect of GPX4 on thyroid cancer tumorigenesis and prognosis.

Methods: Based on The Cancer Genome Atlas (TCGA) and Gene Expression Omnibus (GEO) databases, GPX4 expression was investigated in cancer tissues and adjacent tissues. We determined the biological functions of GPX4-associated differentially expressed genes (DEGs) by using the “clusterProfiler” R package. In addition, the predictive value of GPX4 in thyroid cancer was assessed by using Cox regression analysis and nomograms. Finally, we conducted several *in vitro* experiments to determine the influence of GPX4 expression on proliferation and ferroptosis in thyroid cancer cells.

Results: GPX4 expression was obviously elevated in thyroid cancer tissues compared with normal tissues. Biological function analysis indicated enrichment in muscle contraction, contractile fiber, metal ion transmembrane transporter activity, and complement and coagulation cascades. GPX4 overexpression was associated with stage T3-T4 and pathologic stage III-IV in thyroid cancer patients. Cox regression analysis indicated that GPX4 may be a risk factor for the overall survival of thyroid cancer patients. *In vitro* research showed that knockdown of GPX4 suppressed proliferation and induced ferroptosis in thyroid cancer cells.

Conclusions: GPX4 overexpression in thyroid cancer might play an essential role in tumorigenesis and may have prognostic value for thyroid cancer patients.

## INTRODUCTION

Although thyroid cancer is the most prevalent endocrine malignancy [1, 2], it has a favorable survival outcome, with a 10-year survival rate of more than 90% and a low mortality rate [3–5]. Although endocrine therapy, surgery, radiotherapy, and other technologies continue to improve, 10% of thyroid cancer patients still experience recurrence and metastasis. Once cancer cells metastasize to distant organs, patients have poor prognosis and a markedly increased mortality rate [6, 7]. In the last few years, the survival rates of thyroid cancer patients have notably increased due to improvements in molecular pathology detection and the development of targeted therapies. A number of genes, including *BRAF, RET* and *TERT*, correlate with the tumorigenesis, metastasis and prognosis of thyroid cancer [8, 9]. Unfortunately, these markers are not sufficiently sensitive or accurate, as thyroid cancer patients can have good prognosis even if these genes are mutated. Thus, it is necessary to search for more convincing biomarkers in patients with thyroid cancer.

Ferroptosis was recently discovered to be a pathway of programmed cell death distinct from necrosis, autophagy and apoptosis [10]. Ferroptosis differs from other forms of programmed cell death in two aspects: glutathione peroxidase 4 (GPX4) becomes inhibited, and iron-dependent lipid reactive oxygen species (ROS) accumulate [11]. Various studies have indicated that ferroptosis reveals new prospects for cancer therapy. GPX4 protects cancer cells against oxidative damage, inhibits ferroptosis and can have an oncogenic effect in multiple cancers [12]. However, the underlying role and potential mechanism of GPX4 in thyroid cancer remain unclear.

We first acquired RNA sequencing data and clinical features of thyroid cancer patients from The Cancer Genome Atlas (TCGA). Then, bioinformatics analysis was performed to assess the effects of GPX4-associated differentially expressed genes (DEGs) on thyroid cancer tumorigenesis and prognosis. Next, we conducted correlation analysis of clinicopathological features and GPX4 expression. In addition, we collected clinical specimens and detected differences in GPX4 expression between cancer and paracancerous tissues. Finally, we conducted *in vitro* experiments to investigate whether GPX4 expression affects proliferation and activates ferroptosis in thyroid cancer cells. In summary, GPX4 may have diagnostic and prognostic value, and GPX4 overexpression may be closely related to unfavorable prognosis in patients with thyroid cancer. This research presents GPX4 as an underlying diagnostic and prognostic biomarker and provides novel insight into the pathogenesis of thyroid cancer.

## RESULTS

### Analysis of GPX4 expression in pancancer and thyroid cancer datasets

**Figure 1:**
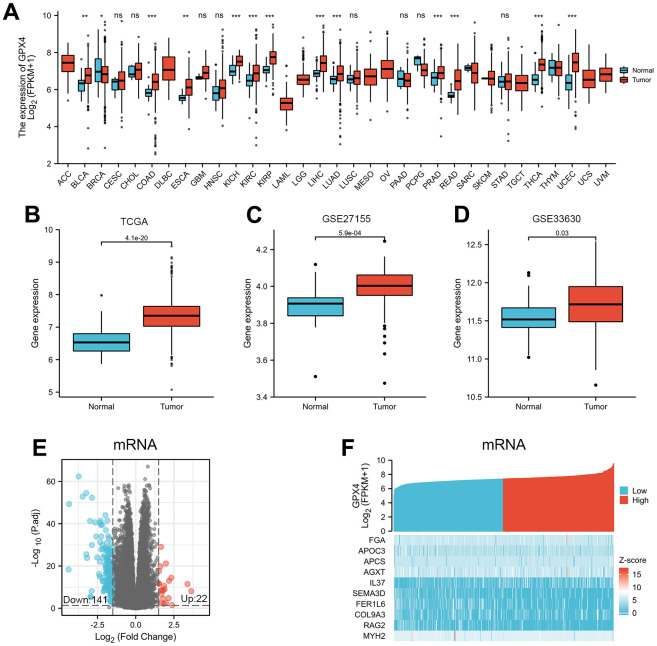
**Differential mRNA expression profiles in thyroid cancer patients stratified by GPX4 level.** (**A**) Comparison of GPX4 expression between different types of cancers and adjacent tissues based on TCGA. ns indicates *p*≥0.05, *p<0.05, **p<0.01, ***p<0.001. GPX4 expression is higher in thyroid cancer tissue than in adjacent normal tissue according to TCGA (**B**) as well as GSE27155 (**C**) and GSE33630 (**D**) from GEO. Differentially expressed mRNAs between the two groups are displayed by volcano plots (**E**) and heatmaps (**F**).

We first evaluated expression of GPX4 mRNA in pancancer data from TCGA. As shown in Figure 1A, the analysis revealed GPX4 expression to be higher in 22 of 33 cancer types (The full names of tumor abbreviation referred to Supplementary Table 1). Interestingly, GPX4 expression was not significantly decreased, except in breast invasive carcinoma. Then, we further validated that GPX4 expression in thyroid cancer tissues was obviously higher than that in normal tissues using TCGA data (Figure 1B, *p*<0.001). In addition, we confirmed the above results in the GEO database. As expected, GPX4 was highly expressed in thyroid cancer according to the GSE27155 (*p*=5.9e-04) (Figure 1C) and GSE33630 (*p*=0.03) (Figure 1D) datasets.

DEGs in tumors are important, as they reflect functional differences in regulation and may be drivers of cancers [13, 14]. Therefore, we divided 510 thyroid cancer patients into two groups according to the median value of GPX4 expression: high and low GPX4 expression groups. Next, mRNA, miRNA, and lncRNA expression was compared between the groups. We regarded 163 mRNAs (22 upregulated and 141 downregulated, Figure 1E), 4 miRNAs (2 upregulated and 2 downregulated, Supplementary Figure 1A), and 68 lncRNAs (3 upregulated and 65 downregulated, Supplementary Figure 1C) as DEGs (absolute value of fold change >1.5, *p*<0.05). Heatmaps were used to illustrate representative DEGs (Figure 1F and Supplementary Figure 1B, 1D).

### GPX4 expression in thyroid cancer tissues and paracancerous tissues

**Figure 2:**
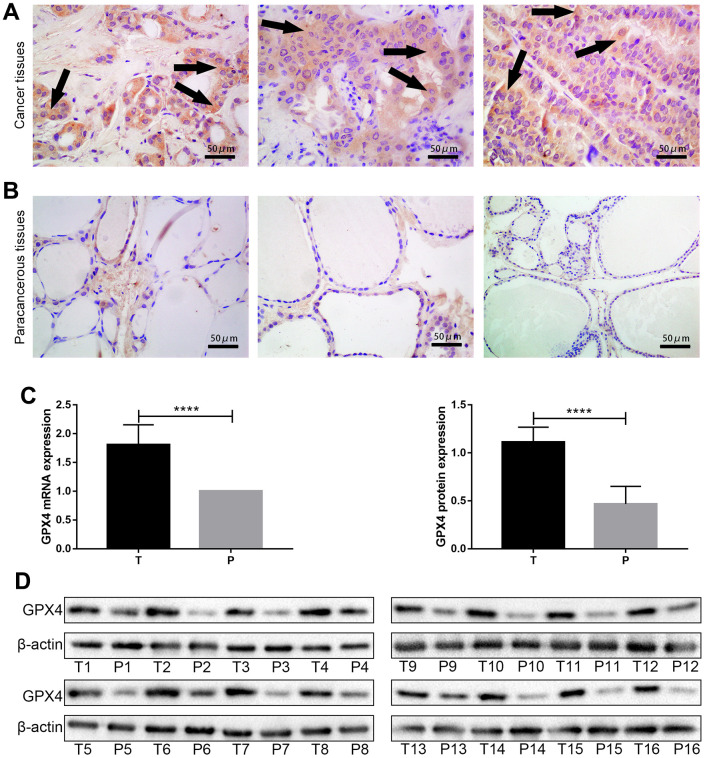
**GPX4 expression in thyroid cancer tissues and paracancerous tissues.** High expression of GPX4 in thyroid cancer tissues (**A**) and low expression of GPX4 in paracancerous tissues (**B**) based on immunohistochemistry (Original magnification×40); scale bars: 50 μm. Expression of GPX4 at the mRNA level (**C**) and protein level (**D**) in different tissues (T, thyroid cancer tissues; P, paracancerous tissues, N=16). ****p<0.0001.

We further confirmed GPX4 expression in thyroid cancer via immunohistochemistry of clinical specimens, and representative images are presented in Figure 2A, 2B. Then, we detected GPX4 expression by qRT-PCR and western blot assays in cancer tissues and corresponding paracancerous tissues. The results showed obviously higher GPX4 expression in cancer tissues than in paracancerous tissues (N=16, Figure 2C, 2D).

### Functional enrichment analyses of GPX4-associated DEGs

**Figure 3:**
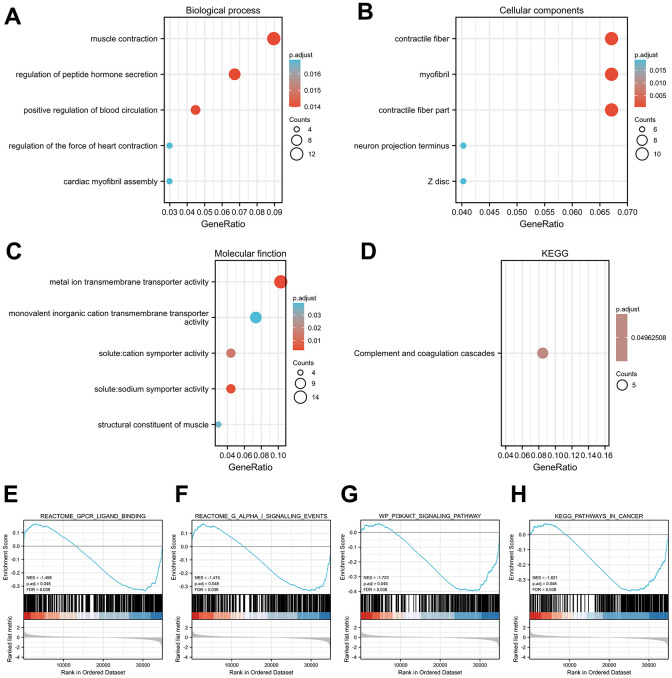
**Functional enrichment analysis results.** Significant Gene Ontology terms of the GPX4-associated DEGs, including BP (**A**), CC (**B**), and MF (**C**). (**D**) Significant KEGG pathways of the GPX4-associated DEGs. Significant GSEA results of GPX4-associated DEGs, including Gpcr ligand binding (**E**), G alpha I signaling events (**F**), PI3KAKT signaling pathway (**G**), and pathways in cancer (**H**).

To assess functional enrichment of GPX4-associated DEGs in thyroid cancer patients, relevant DEGs were enriched using GO analyses in biological process (Figure 3A), cellular component (Figure 3B), and molecular function (Figure 3C) by the “clusterProfiler” R package. The DEGs were only enriched in the KEGG pathways of the complement and coagulation cascades (Figure 3D). Reactome and KEGG pathways were searched by using GSEA, which revealed that Gpcr ligand binding (Figure 3E), G alpha I signaling events (Figure 3F), the PI3KAKT signaling pathway (Figure 3G) and pathways in cancer (Figure 3H) were significantly enriched. These results indicate the potential role of GPX4 in the tumorigenesis of thyroid cancer, especially with regard to signaling-related pathways.

### Correlation between GPX4 expression and clinicopathological features in thyroid cancer patients

**Figure 4:**
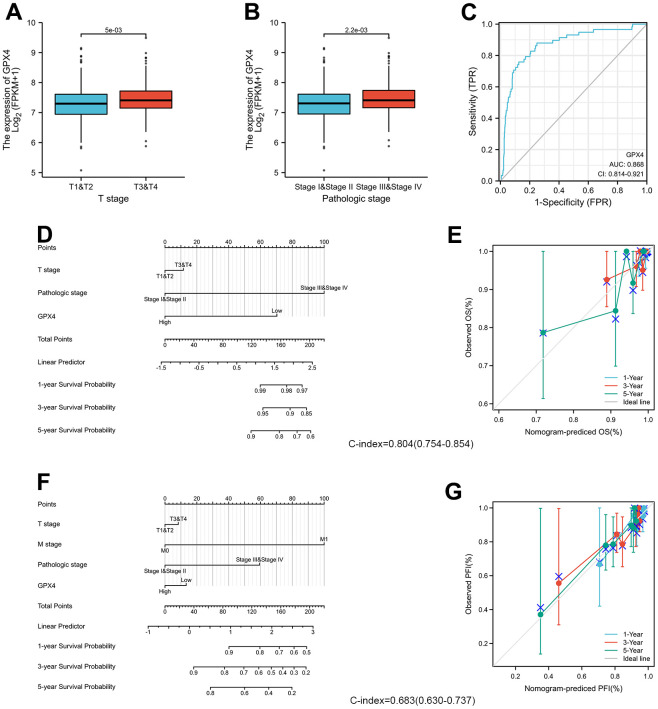
**GPX4 expression and clinicopathological features in thyroid cancer patients.** The Wilcoxon rank sum test was applied to analyze associations of clinical T stage (**A**) and pathologic stage (**B**) with GPX4 expression. (**C**) ROC curve analysis evaluating the diagnostic performance of GPX4 in thyroid cancer. (**D**) Nomogram was built to examine 1-, 3-, and 5-year overall survival based on the risk score model of GPX4 expression. (**E**) Calibration plot verifying the efficiency of the nomogram for overall survival. (**F**) Nomogram was built to examine 1-, 3-, and 5-year progression-free interval based on the risk score model of GPX4 expression. (**G**) Calibration plots verifying the efficiency of the nomogram for progression-free interval.

We examined the clinicopathological features of thyroid cancer patients with differential expression of GPX4. The results indicated that GPX4 expression was significantly higher in patients at stage T3-T4 disease (Figure 4A) and pathologic stage III-IV disease than in those at other disease stages (Figure 4B). Furthermore, we used an ROC curve to explore the clinical diagnostic value of GPX4 in thyroid cancer. The area under the curve (AUC, 0.868) showed that GPX4 has high sensitivity and specificity in thyroid cancer diagnosis (Figure 4C).

Moreover, the predictive value of GPX4 for clinical outcomes was evaluated by using Cox regression analysis. As shown in Table 1, GPX4 was determined to be an independent risk factor for overall survival based on univariate Cox regression (HR: 0.29, *p*=0.032) and multivariate Cox regression (HR: 026, *p*=0.021), though it did not have any obvious predictive value for the progression-free interval. In particular, clinical T and M stages and pathologic stage displayed good predictive advantages in Cox regression analyses.

**Table 1:** Cox regression analysis for clinical outcomes in thyroid cancer patients.

**Characteristics**	**Total(N)**	**HR for overall survival (95% CI)**		**HR for progression-free interval (95% CI)**	
		**Univariate**	**Multivariate**	**Univariate**	**Multivariate**
**T stage**	508	2.97(1.03-8.55) *	1.70(0.44-6.62)	2.45(1.42-4.24) **	1.64(0.52-5.14)
T1&T2	310				
T3&T4	198				
**N stage**	460	1.43(0.47-4.37)		1.66(0.94-2.93)	1.03(0.48-2.22)
N0	229				
N1	231				
**M stage**	295	4.26(0.910-19.96)	2.12(0.45-10.07)	7.31(2.78-19.20) ***	2.88(0.67-12.29)
M0	286				
M1	9				
**Pathologic stage**	508	7.19(2.31-22.35) ^***^	7.54(2.43-23.45) ***	2.60(1.52-4.44) ***	2.60(1.52-4.44) ***
StageI&StageII	338				
StageIII&StageIV	170				
**Gender**	510	1.96(0.71-5.43)		1.70(0.98-2.95)	1.23(0.55-2.71)
Female	371				
Male	139				
**GPX4**	510	0.29(0.09-0.90) *	0.26(0.09-0.82) *	0.98(0.57-1.67)	
Low	255				
High	255				

HR, hazard ratio; CI, confidence interval; *p<0.05; **p<0.01; ***p<0.001.

The prognostic factors indicated to be statistically significant were used to establish a prognostic nomogram, and we further generated calibration curves to verify the efficiency of this nomogram. Clinical T stage, pathologic stage, and GPX4 were included to predict overall survival; the C-index of the nomogram was 0.804 (Figure 4D). Clinical T and M stage, pathologic stage and GPX4 were included to predict the progression-free interval; the C-index of the nomogram was 0.683 (Figure 4F). The corresponding calibration curves all showed that the two nomograms had satisfactory predictive ability for clinical outcomes, except that the 5-year prediction for overall survival was slightly inaccurate (Figure 4E, 4G).

### Knockdown of GPX4 inhibits proliferation in thyroid cancer cells

**Figure 5:**
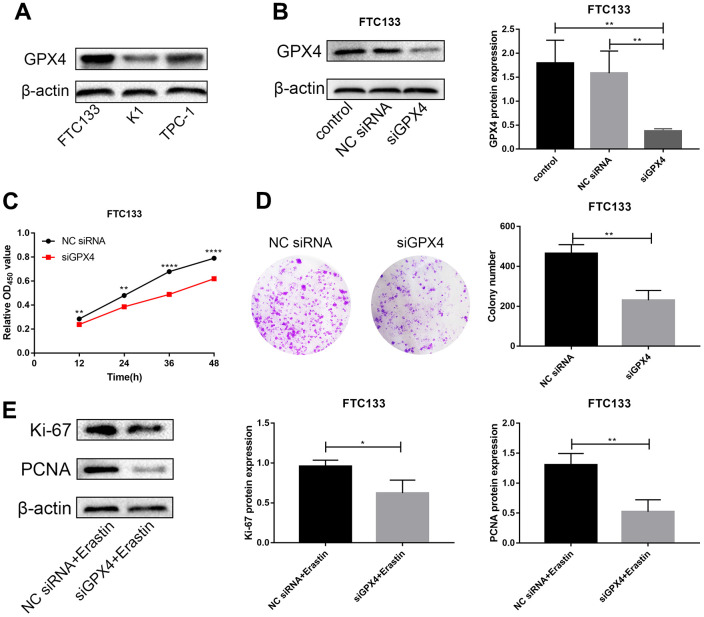
**Knockdown of GPX4 inhibits proliferation in thyroid cancer cells.** (**A**) GPX4 expression levels in FTC133, K1 and TPC-1 cells by western blot analysis. (**B**) GPX4 expression in FTC133 cells transfected with control, NC siRNA, or siGPX4 was confirmed by western blot analysis. (**C**) A CCK-8 assay was used to evaluate the viability of FTC133 cells transfected with NC siRNA/siGPX4. (**D**) Colony formation assay demonstrated the proliferation ability of FTC133 cells transfected with NC siRNA/siGPX4. (**E**) Ki-67 and PCNA protein expression in FTC133 cells transfected with NC siRNA/siGPX4 under erastin treatment.

We first detected protein expression of GPX4 in three thyroid cancer cell lines (FTC133, K1 and TPC-1), with FTC133 cells exhibiting higher GPX4 protein expression than the other cell lines (Figure 5A). Therefore, we selected FTC133 cells to conduct subsequent functional tests. First, siRNA targeting GPX4 was transfected into FTC133 cells, and western blotting was applied to examine the efficiency of knockdown (Figure 5B). Western blot analysis demonstrated a significant reduction in GPX4 expression after transfection with siGPX4. CCK-8 assays were used to assess cellular viability to investigate the effect of GPX4 on the proliferation of thyroid cancer cells, and the results showed that GPX4 knockdown markedly decreased FTC133 cell viability (Figure 5C). Similarly, colony formation experiments demonstrated that inhibition of GPX4 suppresses the proliferation of FTC133 cells (Figure 5D). Erastin (M2679, Sigma, St Louis, USA), a classic ferroptosis inducer, can activate the ferroptosis signaling pathway in cells [15]. Thus, to further study the specific mechanism responsible for inhibiting proliferation, expression of proliferation markers (Ki-67 and PCNA) was evaluated in FTC133 cells transfected with NC siRNA/siGPX4 after treatment with erastin. The results verified that GPX4 knockdown obviously inhibited protein expression of Ki-67 and PCNA (Figure 5E), as well as proliferation in thyroid cancer cells.

### Knockdown of GPX4 activates ferroptosis in thyroid cancer cells

**Figure 6:**
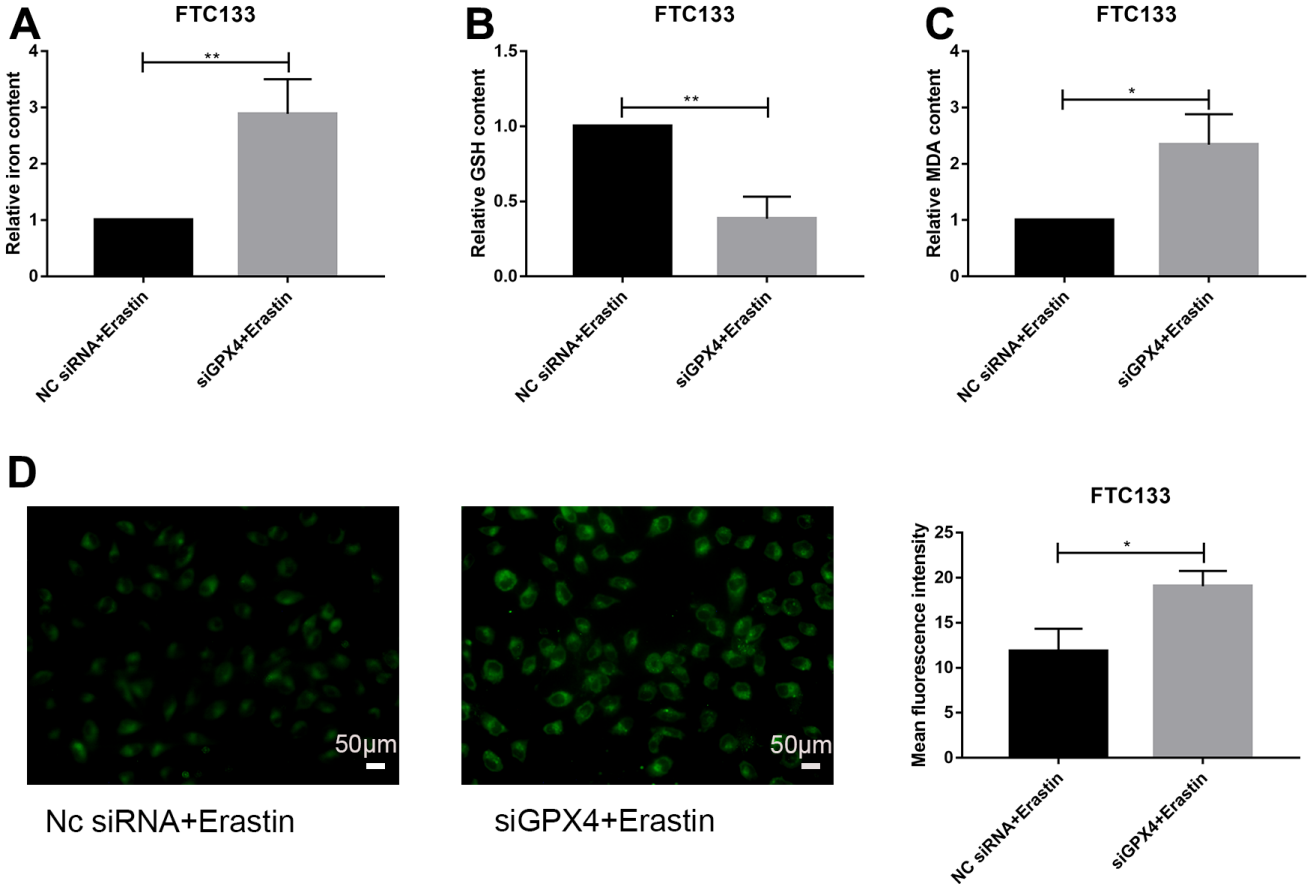
**Knockdown of GPX4 activates ferroptosis in thyroid cancer cells.** Relative iron (**A**), GSH (**B**) and MDA (**C**) contents in FTC133 cells transfected with NC siRNA/siGPX4 after treatment with erastin. (**D**) Detection of lipid ROS levels in FTC133 cells transfected with NC siRNA/siGPX4 after treatment with erastin; scale bars: 50 μm. *p<0.05, **p<0.01.

We further investigated the regulatory effect of GPX4 knockdown on ferroptosis under erastin treatment. Indicators related to ferroptosis were examined, including iron content, GSH, MDA and lipid ROS assays. The results showed that knockdown of GPX4 increased the iron content of FTC133 cells treated with erastin (Figure 6A); the MDA content was also markedly increased after erastin treatment (Figure 6C). However, the GSH content was significantly decreased (Figure 6B). Finally, lipid ROS experiments showed that GPX4 knockdown induced lipid peroxidation in FTC133 cells (Figure 6D). Thus, we infer that knockdown of GPX4 obviously activates the ferroptosis signaling pathway in FTC133 cells.

## DISCUSSION

Thyroid cancer, local recurrence and metastasis of which result in death, has a very high incidence and has become the fifth most common cancer among women in the United States [5, 16]. Therefore, research on precise diagnostic and prognostic biomarkers is urgent. Ferroptosis has great advantages in tumor therapy [11]. GPX4, identified as a central regulator of ferroptosis, reduces lipid hydroperoxides to nontoxic lipid alcohols, thereby protecting cells against damage. Evidence indicates that GPX4 inhibitors have specific lethality in tumor cells through ferroptosis [17, 18], but no associated research has been conducted in thyroid cancer.

The purpose of this study was to examine the gene expression profiles, clinicopathological characteristics, and clinical significance of GPX4 in thyroid cancer patients by analyzing TCGA data. We observed that GPX4 expression was prominently increased in thyroid cancer, and the result was confirmed by the GEO database and in thyroid cancer and paracancerous tissues. As abnormal gene expression is closely associated with a range of pathological conditions [19], GPX4-associated DEGs of the high GPX4 expression group and the low GPX4 expression group were explored. In this study, we identified 22 upregulated and 141 downregulated DEGs among GPX4-related mRNAs. Next, functional enrichment analysis was carried out on the GPX4-associated DEGs. We found that these DEGs were specifically enriched in muscle contraction, contractile fiber, metal ion transmembrane transporter activity, and complement and coagulation cascades. GSEA suggested that these DEGs are associated with the signaling pathways and tumorigenesis of malignant tumors.

The clinical significance of GPX4 in thyroid cancer is another focus of attention. Based on ROC curve analysis, GPX4 seems to be a reliable biomarker for thyroid cancer diagnosis. Furthermore, we found that GPX4 overexpression was associated with several clinicopathological features: stage T3-T4 disease and pathologic stage III-IV disease. Further Cox regression and nomogram analyses demonstrated that GPX4 accurately predicts the clinical outcomes of thyroid cancer. Patients with GPX4 overexpression displayed obviously worse overall survival than those with low GPX4 expression. The nomogram analysis also supported the above findings. The predictive performance of GPX4 suggests that it might be regarded as a universal prognostic biomarker for thyroid cancer.

Friedmann Anageli et al. provided direct genetic evidence that cell death caused by knockout of GPX4 is related to pathological ferroptosis [20]. Therefore, to explore the influence of GPX4 in thyroid cancer cells, we focused our attention on FTC133 cells because of their higher expression of GPX4. Two other important reasons are that follicular thyroid cancer (FTC) is more aggressive than papillary thyroid cancer (PTC) and more difficult to diagnose unless paraffin pathological sections are examined [21–23]. CCK-8 and colony formation assays showed that low GPX4 expression in FTC133 cells markedly restrained their proliferation; the specific mechanism is that GPX4 inhibits Ki-67 and PCNA protein expression. The Ki-67 protein is considered a proliferation marker for human tumor cells. Ki-67 acts during both interphase and mitosis [24, 25]. The PCNA protein is essential for tumor cell proliferation and participates in a variety of processes of DNA metabolism [26, 27]. Thus, decreased protein expression of PCNA and Ki-67 might inhibit tumor cell proliferation.

Ferroptosis is a type of iron-dependent oxidative cell death characterized by a high iron content [28, 29], a high MDA level [30], and GSH depletion [31]. In addition, ferroptosis is induced by accumulation of reactive lipid ROS [32]. Knockdown of GPX4 obviously activated the ferroptosis signaling pathway under erastin treatment. In fact, recent studies have demonstrated that GPX4 is inhibited by various drugs or molecules that induce ferroptosis [33–35]. Levels of hydroperoxide derived from complex lipids can be decreased by GPX4, a monomeric glutathione peroxidase [36]. The key event of ferroptosis is enhancement of lipid peroxidation induced by GPX4 inhibition [37]. GPX4 protects cells against oxidative damage, which is harmful to multiple cancers, including thyroid cancer. The glutathione (GSH)-GPX4 system is considered a main protective system that prevents ferroptosis in cells [37, 38]. Therefore, GPX4 is identified as a central regulator of ferroptosis.

Although we discovered the predictive value of GPX4 in thyroid cancer patients and uncovered its potential pathogenic mechanism, there are still some limitations to our study. We mainly focused on analyzing RNA sequencing data in TCGA and failed to supply relevant clinical survival information. Thus, to better explain the predictive value of GPX4, we will collect clinical data and focus on the prognostic effect of GPX4 expression in thyroid cancer in the future.

## CONCLUSIONS

In summary, GPX4 is overexpressed in thyroid cancer, and overexpression of GPX4 correlates with tumor progression. Therefore, GPX4 may be considered an independent risk factor for the overall survival of thyroid cancer patients. Furthermore, we determined that GPX4 facilitates proliferation and inhibits ferroptosis in thyroid cancer cells. The above results suggest that GPX4 plays an essential role in tumorigenesis, tumor progression and malignant behavior. Thus, GPX4 may constitute a biomarker for thyroid cancer diagnosis and prognosis evaluation, and targeting GPX4 might be an effective treatment for thyroid cancer.

## MATERIALS AND METHODS

### Data source

TCGA, a free data site of the human cancer genome project, offers data on 33 types of cancer, including clinical and pathological information, to researchers. RNA sequencing data and matching clinical features of thyroid cancer patients were acquired from TCGA. The GSE27155 and GSE33630 datasets were acquired from Gene Expression Omnibus (GEO), a comprehensive gene expression database.

### Sample collection

Thyroid cancer and adjacent tissues from 16 patients were collected and immediately preserved in liquid nitrogen. All samples were collected with the written informed consent of the individual participants involved in this research. The research was performed in a manner consistent with the Declaration of Helsinki.

### GPX4-associated DEGs and functional enrichment analyses

A total of 510 thyroid cancer patients in TCGA were enrolled. The R package “DESeq2” was used to determine DEGs, and we set the thresholds as a log-fold change greater than 1.5 and a *p value* lower than 0.05. Heatmaps and volcano plots were generated using the “pheatmap” and “EnhancedVolcano” R packages. Gene Ontology (GO) terms for the biological process (BP), molecular function (MF) and cellular component (CC) categories, as well as Kyoto Encyclopedia of Genes and Genomes (KEGG) pathways, were identified by using the “clusterProfiler” R package, which was also used to visualize the gene set enrichment analysis (GSEA) results.

### Immunohistochemistry

Human thyroid tumor specimens were acquired from the Department of Pathology. Tumor sections (3 μm) were incubated with the primary antibody anti-GPX4 (1:100, Proteintech [14432-1-AP], China) overnight at 4° C. The next day, the sections were incubated with goat anti-rabbit Envision System Plus-HRP for 30 minutes at room temperature. The sections were rinsed three times in phosphate-buffered saline (PBS) for 10 minutes each, and then diaminobenzidine (DAB) was applied, followed by incubation for 2 minutes. The tumor sections were dehydrated and mounted after counterstaining with Mayer hematoxylin. Finally, the sections were visualized using an Olympus microscope at a magnification of ×40.

### Quantitative real-time PCR analysis

Total RNA was extracted from clinical specimens using an RNA extraction kit (Accurate Biology, AG21017). Evo M-MLV RT Premix (Accurate Biology, AG11706) and SYBR Premix Ex TaqTM (Accurate Biology, AG11702) were used to prepare samples for RT-qPCR, which was conducted using a Rotor-Gene Q instrument (Qiagen, Germany). We calculated the relative expression levels of target genes based on the 2^-ΔΔCT^ method. The primers used were as follows: human β-actin Forward: 5’– GGCACCCAGCACAATGAA–3’; human β-actin Reverse: 5’– TAGAAGCATTTGCGGTGG–3’; human GPX4 Forward: 5’– ATCGACGGGCACATGGTTAA–3’; human GPX4 Reverse: 5’– CAGGATCCGCAAACCACACT–3’.

### Western blot assay

Western blot assays were used to detect high or low levels of protein expression. The thyroid cancer cells used in experiments or solid tumor tissues were dissolved to radioimmunoprecipitation assay (RIPA) lysis buffer. After resolving proteins on 10% SDS-PAGE gels, the proteins were transferred onto PVDF membranes and incubated at 4° C overnight with the primary antibodies: anti-GPX4 (1:500, Proteintech, China), anti-Ki-67 (1:1000, Proteintech, China), and anti-PCNA (1:1000, Proteintech, China). Next, the PVDF membranes were incubated with peroxidase-conjugated secondary antibody (1:3000, Proteintech, China) for 2 hours at room temperature. The western blotting results were calculated by using ImageJ software.

### Clinicopathological features and clinical significance of GPX4 expression in thyroid cancer

Clinicopathological features were compared by using the Wilcoxon rank sum test or Pearson’s chi-square test. The predictive accuracy of GPX4 for thyroid cancer diagnosis was evaluated by receiver operating characteristic (ROC) curve analysis. Information on the clinical outcomes of overall survival and progression-free interval were obtained from TCGA. Univariate and multivariate Cox regression analyses were used to analyze prognosis. We employed the R package “rms” to construct nomograms and calibration diagrams. R statistical software (version 3.6.3; https://www.r-project.org/) was used for all statistical analyses.

### Cell culture and transfection

The human thyroid cancer cell lines FTC133, K1, TPC-1 were obtained from the cell bank of the Chinese Academy of Sciences (Shanghai, China) and cultivated in Dulbecco’s modified Eagle’s medium (DMEM) supplemented with 10% fetal bovine serum (FBS). All cells were cultivated at 37° C in a 5% CO_2_ incubator.

An siRNA targeting GPX4 was constructed by GenePharma (Shanghai, China). The sequences were as follows: sense: 5’–CAGGGAGUAACGAAGAGAUTT–3’ and antisense: 5’–AUCUCUUCGUUACUCCCUGTT–3’. Transfection steps were conducted following the manufacturer’s protocols, and Lipofectamine™3000 (Invitrogen, USA) was used.

### Cell viability assay

To evaluate cancer cell viability, cells in logarithmic growth phase were digested by using trypsin and then evenly added to a 96-well plate (density: 5×10^3^ cells per well). Each group was assayed in 6 duplicate wells. The plate was placed in an incubator at 37° C in a 5% CO_2_ environment. Cell Counting Kit-8 (CCK-8; Meilun, Dalian, China) solution (10 μl) was added to each well, and the 96-well plate was incubated at 37° C for 1 hour. A microplate reader (Thermo Fisher Scientific, Shanghai, China) was used to measure absorbance at 450 nm daily for two days.

### Colony formation assay

A total of 1000 cells were placed in a 6-well plate and incubated at 37° C in a 5% CO_2_ environment. After 1 week, the medium was discarded. PBS was used to lightly wash away the residual medium. The cells were stained with 0.2% dissolved crystal violet after fixation with methanol for 30 minutes, and the number of colonies containing more than 30 cells was counted.

### Iron content detection

Iron content was determined by using an Intracellular Iron Colorimetric Assay Kit (Applygen Technology Inc. Beijing, China). In detail, cells were collected with chilled PBS and centrifuged at 12000 rpm for 10 minutes. The supernatant was used for subsequent tests. All operations were executed according to the manufacturer’s instructions, and iron contents were calculated by measuring absorbance at 550 nm.

### Measurement of glutathione (GSH) and malondialdehyde (MDA) content

GSH is an antioxidant, and MDA is an end product of lipid peroxidation; both are markers of oxidative stress. Commercial GSH and MDA assay kits were purchased from Nanjing Jiancheng Bioengineering Institute (Nanjing, China). Cell samples were treated as described in “iron content detection” above. We followed the manufacturer’s instructions for all operations. Individual contents of GSH and MDA were assessed at 405 and 532 nm, respectively, using a 96-well plate. The total protein concentration was determined according to the instructions of BCA Protein Assay Kit (KGP902, KeyGEN Bio TECH, China).

### Lipid ROS assay

Lipid ROS, considered one of the most important markers of ferroptosis, was measured by using Liperfluo (#L248, Dojindo, Kumamoto, Japan). We seeded cells in 24-well plates, pretreated them with erastin-containing medium for 24 hours, and incubated them for 30 minutes in the dark with Liperfluo at a working concentration of 1 μM. PBS was then used to wash the cells three times. Finally, a fluorescence microscope (Olympus, Tokyo, Japan) was used to acquire images at a magnification of ×40.

### Statistical analysis

All experiments were repeated at least 3 times. The level of GPX4 expression based on TCGA data was visualized by box plots. The Wilcoxon rank sum test and Pearson’s chi-square test were performed to analyze the association between clinical features and GPX4 expression in thyroid cancer; the former was applied for continuous variables and the latter for rank variables. Cox analysis was used to explore the predictive value of GPX4 and to screen prognostic factors. Student’s tests were used to compare values between clinical samples and *in vitro* experiments. Statistical significance was considered as p value less than 0.05.

## Supplementary Material

Supplementary Figure 1

Supplementary Table 1
