# Supplementary material for: Increased expression of GPX4 promotes the tumorigenesis of thyroid cancer by inhibiting ferroptosis and predicts poor clinical outcomes
Source: Aging (Albany NY). 2023 Jan 9;15(1):230–45. doi: 10.18632/aging.204473 (PMC9876627; doi:10.18632/aging.204473)
Supplement: Supplementary Figure 1 [file aging-15-204473-s001.pdf]

SUPPLEMENTARY FIGURE

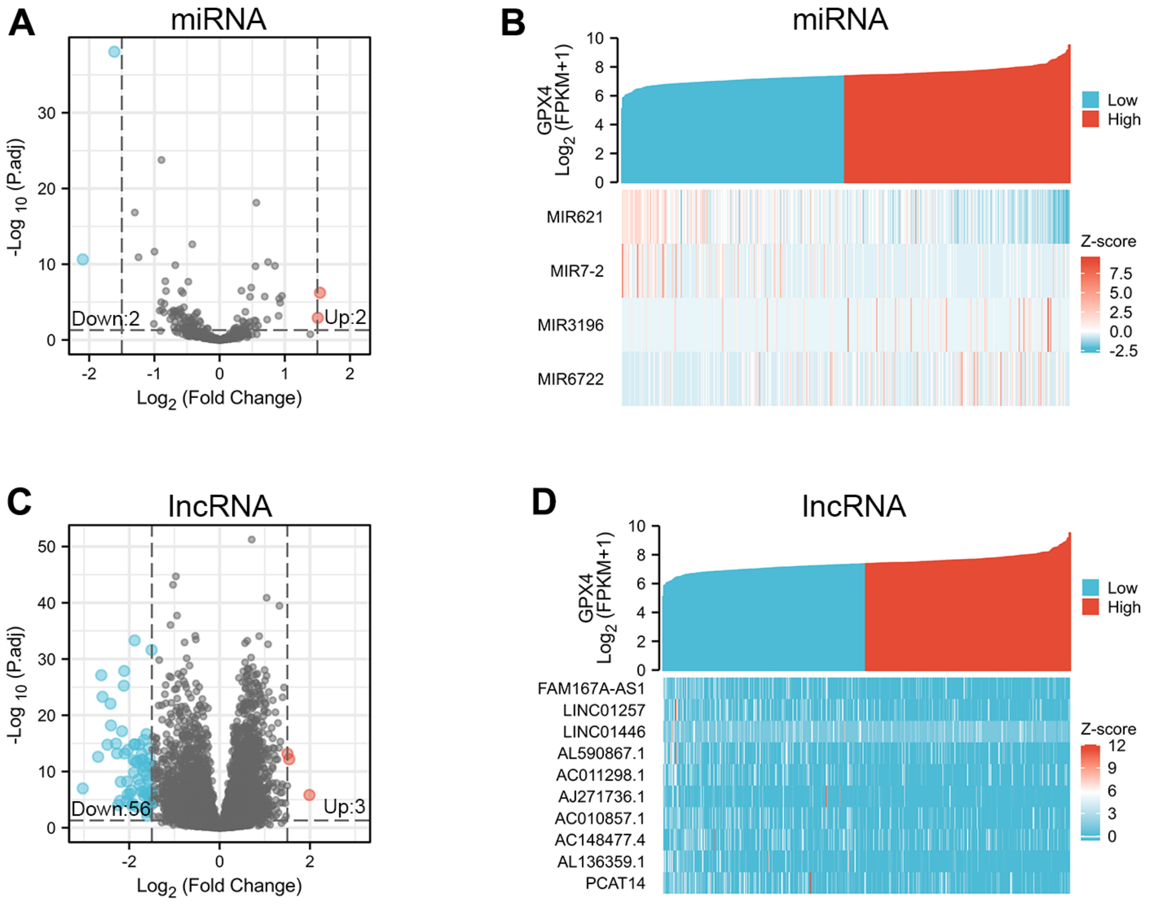

Supplementary Figure 1. Differential non-coding RNA expression profiles in thyroid cancer patients stratified by GPX4 levels. Expression profiles of miRNAs in two groups are presented by volcano plot (**A**) and heatmap (**B**). Expression profiles of lncRNAs in two groups are presented by volcano plot (**C**) and heatmap (**D**).
